# Supplementary material for: 4D-ONIX for reconstructing 3D movies from sparse X-ray projections via deep learning
Source: Commun Eng. 2025 Mar 21;4:54. doi: 10.1038/s44172-025-00390-w (PMC11928503; doi:10.1038/s44172-025-00390-w)
Supplement: Supplementary file 1 — Supplementary Material [file 44172_2025_390_MOESM1_ESM.pdf]

Supplementary material: 4D-ONIX for reconstructing 3D movies from sparse X-ray projections via deep learning

Yuhe Zhang[1,*], Zisheng Yao[1], Robert Klöfkorn[2], Tobias Ritschel[3], and Pablo Villanueva-Perez[1]

[1]Synchrotron Radiation Research and NanoLund, Lund University, Box 118, 221 00, Lund, Sweden
[2]Center for Mathematical Sciences, Lund University, Box 117, 221 00, Lund, Sweden
[3]University College London, WC1E 6BT London, UK

*yuhe.zhang@sljus.lu.se

Abstract

This document provides supplementary information for "4D-ONIX for reconstructing 3D movies from sparse X-ray projections via deep learning." In this material, we report supplementary figures and descriptions to complement the main article.

Supplementary Note 1: Reconstruction for reproducible processes with variable number of experiments for simulated water droplet collision

In this section, we investigate the performance of 4D-ONIX by training it with multiple experiments of reproducible processes. Specifically, we randomly generated 16 projection pairs in a 180° range from a simulated droplet collision dataset. The dataset comprises 75 timestamps, which capture the centered collision between two water droplets. The angle between the two projections within each pair is 23.8°. Here, we refer to each unique projection pair as an "experiment".

We trained 4D-ONIX using 1, 2, 4, 8, and 16 experiments separately. For the 16 experiments, the φ_1 angles (azimuthal angles of the first projections, see Figure 2 of the main article) were: 0°, 2°, 13°, 16°, 26°, 28°, 43°, 52°, 64°, 74°, 87°, 95°, 102°, 115°, 130°, 144°. Every second experiment was used for the training with eight experiments. For the training with four experiments, every fourth experiment was employed. The first and the ninth experiments were used for the training with two experiments. All experiments were used for the training with 16 experiments, while only the first experiment was used for the single-experiment training. The precise angles used in the training are detailed in Table 1. Please note that these angles are unknown to 4D-ONIX during the training process. The results were obtained after 800, 800, 800, 400, and 200 epochs for the training with 1, 2, 4, 8, and 16 experiments, respectively, Throughout all training sessions, we set an initial learning rate of 0.0001 and decayed the learning rate by 0.1 in the middle of the total epochs for each training. The rest of the model parameters are the same as those used in the main paper.

We evaluated the performance of the 4D reconstructions reconstructed using different numbers of experiments. The performance evaluation calculates the difference between the 4D-ONIX output and the ground truth. Please note that the ground truth is never shown to the 4D-ONIX model at any stage, and it is used only for evaluation. First, we calculated the 4D Mean Squared Error (MSE) and the Dissimilarity Structure Similarity Index Metric (DSSIM) [1] between the ground truth and the 4D-ONIX reconstructions, as shown in Table 1. DSSIM is defined as $\mathrm{DSSIM} = (1 - \mathrm{SSIM})/2$, where the Structure Similarity Index Metric (SSIM) was calculated using the scikit-image package [2] in Python. Next, we calculated the distribution of 3D MSE and DSSIM with time, as shown in Figure 1**a, b**. The spatial resolution of the reconstructions with 16 experiments was evaluated using Fourier Shell Correlation (FSC) and Fourier Ring Correlation (FRC), which measure the normalized cross-correlation of two images in Fourier space over shells or rings, providing resolution estimates in 3D and 2D, respectively [3, 4]. The

Supplementary Table 1: Comparison of 4D-ONIX reconstructions trained with different numbers of experiments for reproducible processes. The yellow, green, blue, and brown lines represent the additional projections introduced as the number of experiments increases.

# Experiments	1	2	4	8	16
MSE ×10^{-4}	8.10	5.74	3.54	2.68	2.57
DSSIM ×10^{-3}	6.31	4.95	2.85	2.26	2.26
Projection angles	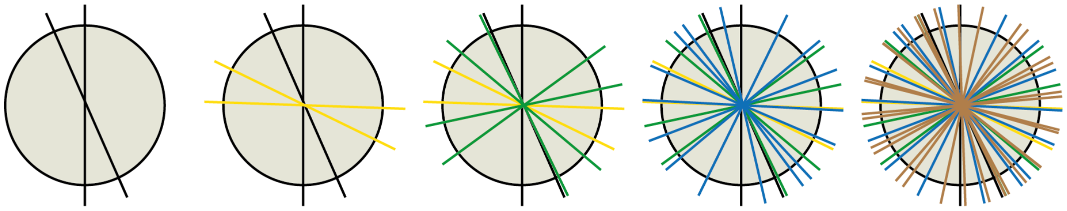				
φ_1 angles	0°	0°,64°	0°,26°,64°,102°	0°,13°,26°,43°, 64°,87°,102°, 130°	0°,2°,13°,16°,26°, 28°,43°,52°,64°, 74°,87°,95°,102°, 115°,130°,144°

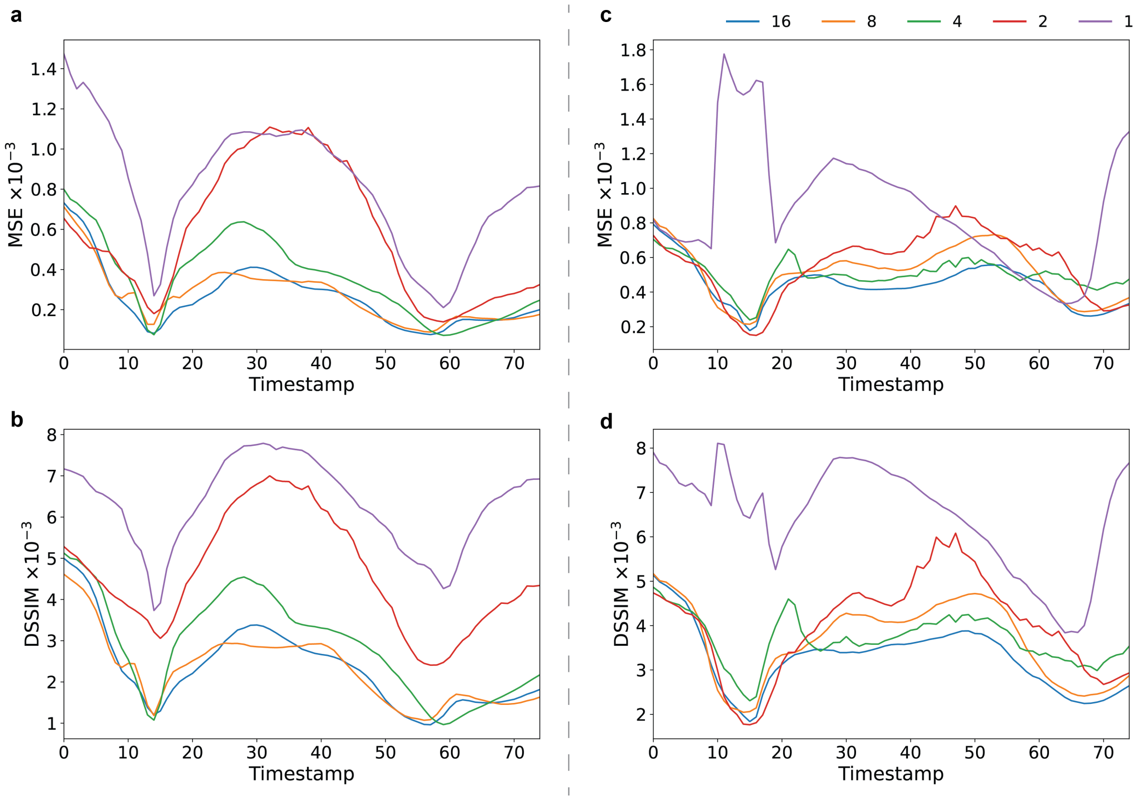

Supplementary Figure 1: Comparison of error distribution with time for 4D-ONIX reconstructions trained with 16 (blue), 8 (orange), 4 (green), 2 (red), and 1 (purple) experiments for a reproducible process (**a, b**) and a quasi-reproducible process (**c,d**). **a** Mean Squared Error (MSE) and **b** Dissimilarity Structure Similarity Index Metric (DSSIM) distribution with time for 4D-ONIX reconstructions trained with different numbers of experiments for a reproducible process. **c** MSE and **d** DSSIM distribution with time for 4D-ONIX reconstructions trained with different numbers of experiments for a quasi-reproducible process. The MSE and DSSIM were computed by comparing the difference between the 4D-ONIX reconstructions and the simulated 3D ground truth at each time point.

results of three example timestamps at different stages of the collision are shown in Figure 2. The FRC was calculated over the trained projection and the unseen projection (perpendicular to the beam plane) separately. The reconstruction results trained with 1, 2, 4, 8, and 16 experiments, along with the ground truth, are presented in Figure 3, Supplementary Movie 1, and Supplementary Movie 2.

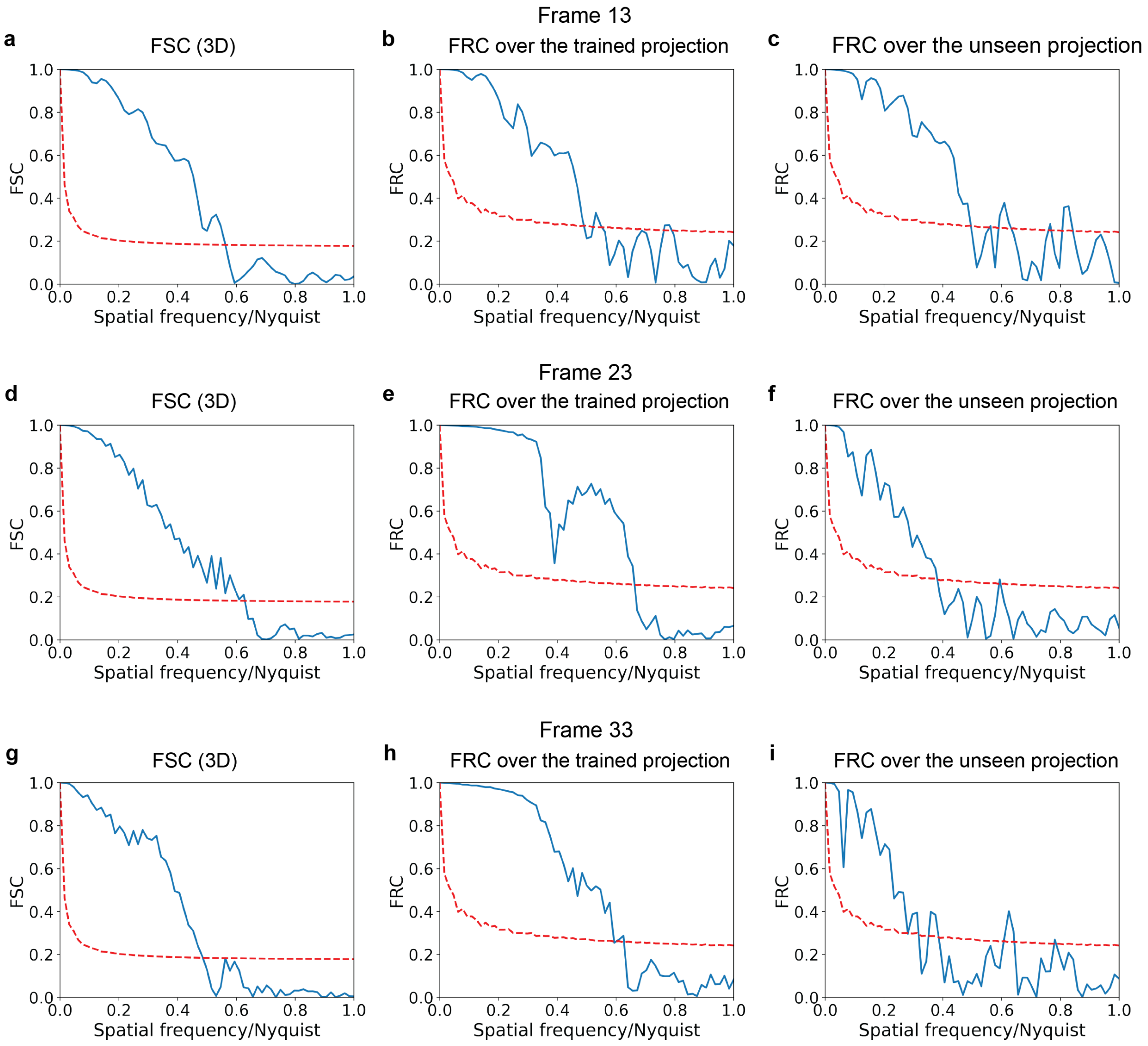

Supplementary Figure 2: Demonstration of the retrieved resolution for the 4D-ONIX reconstructions trained with 16 experiments of reproducible processes. The blue curves represent the 3D Fourier Shell Correlation (FSC) and 2D Fourier Ring Correlation (FRC) across different spatial frequencies, while the red dashed curves indicate the half-bit criterion. Three example timestamps are shown. **a** shows the FSC between the 4D-ONIX reconstruction and the 3D ground truth for timestamp 13, which gives a resolution of around 3.5 voxels. **b** and **c** show for the same timestamp the 2D FRC for the seen projection and the unseen projection, respectively, which correspond to a resolution of 4 pixels for both. **d** the FSC for timestamp 23, which gives a resolution of around 3 voxels. **e** and **e** the 2D FRC for the seen projection and the unseen projection for timestamp 23, which corresponds to a resolution of 3 pixels and 5 pixels, respectively. ‘ **g** the FSC for timestamp 33, which gives a resolution of 4 voxels. **h** and **i** the 2D FRC for the seen projection and the unseen projection for timestamp 33, which corresponds to a resolution of 3 pixels and 7 pixels, respectively.

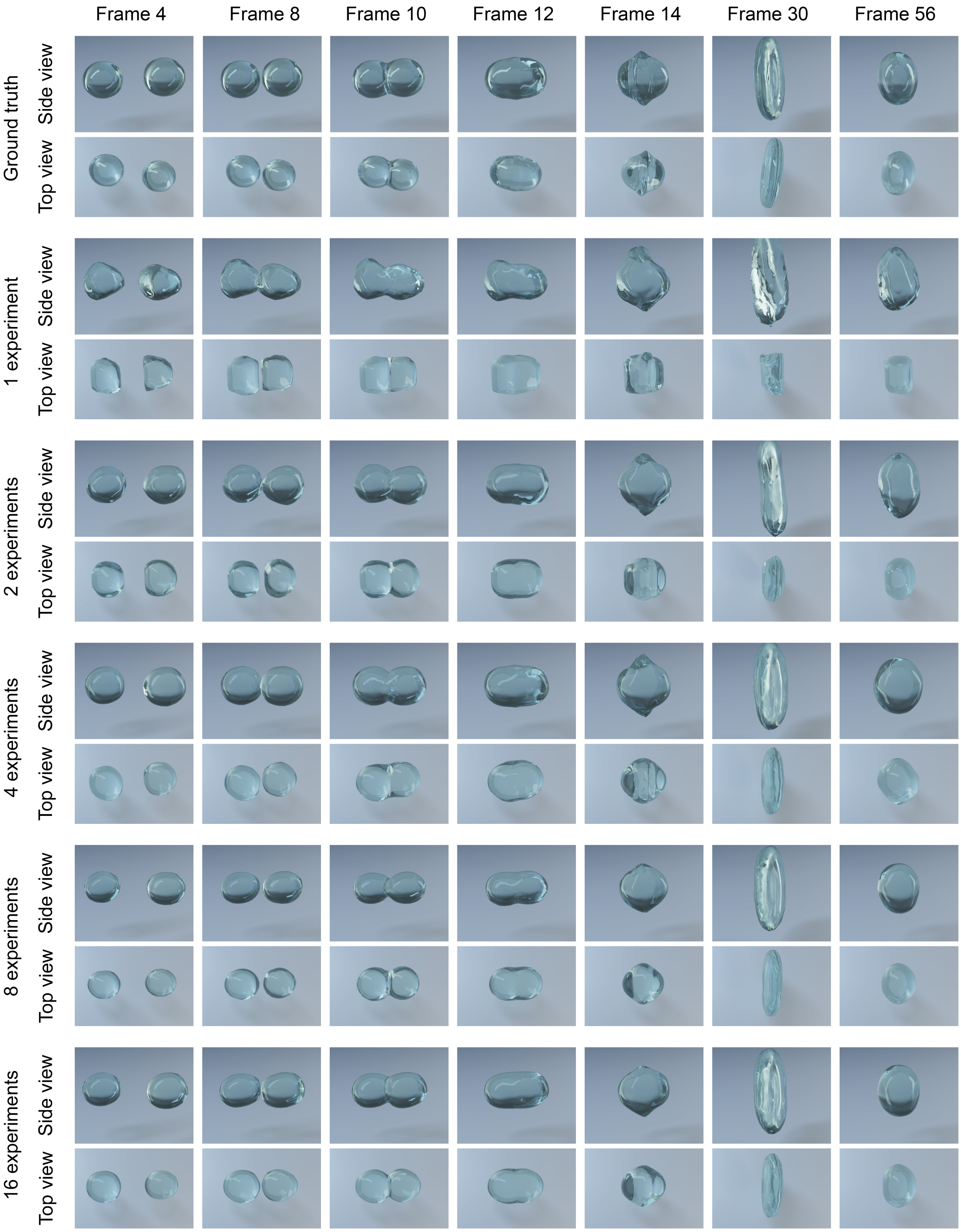

Supplementary Figure 3: Demonstration of 4D-ONIX reconstructions trained with different numbers of experiments for reproducible processes. For the selected timestamps, the 3D rendering from the side and top views of the ground truth and the 4D-ONIX reconstructions using different numbers of experiments are presented.

Supplementary Note 2: Reconstruction for quasi-reproducible processes with a variable number of experiments for simulated water droplet collision

In this section, we assessed the performance of 4D-ONIX by training it with multiple experiments involving quasi-reproducible processes. We conducted 16 simulated droplet collision experiments, each comprising 75 timestamps depicting the collision of two droplets with a 10% variation in droplet size and collision velocity. From each simulation experiment, we generated a projection pair. The angles of the projections were the same as the ones used for the reproducible processes, and the angle between the two projections remains consistent at 23.8°.

We trained 4D-ONIX separately using 1, 2, 4, 8, and 16 experiments and assessed the performance of the 4D reconstructions obtained with different numbers of experiments. The training parameters are the same as reported in the previous section. The 4D MSE and the DSSIM between the ground truth and the 4D-ONIX reconstructions are shown in Table 2. Next, we calculate the distribution of 3D MSE and DSSIM with time, as shown in Figure 1 **c, d**. Examples of the FSC and FRC curves for three timestamps at different stages of the collision are shown in Figure 4. Same as before, the FRC was calculated over the trained projection and the unseen projection (perpendicular to the beam plane) separately. Figure 5, Supplementary Movie 3, and Supplementary Movie 4 show the ground truth and the reconstruction results trained with 1, 2, 4, 8, and 16 experiments.

Supplementary Table 2: Comparison of 4D-ONIX reconstructions trained with different numbers of experiments for quasi-reproducible processes.

# Experiments	φ_1 angles	MSE $\times 10^{-4}$	DSSIM $\times 10^{-3}$
1	0°	8.64	6.46
2	0°, 64°	5.45	4.05
4	0°, 26°,102°,64°	4.84	3.67
8	0°, 13°,26°, 43°, 64°, 87°,102°, 130°	4.98	3.66
16	0°, 2°, 13°, 16°, 26°, 28°, 43°, 52°, 64°, 74°, 87°, 95°, 102°, 115°, 130°, 144°	4.29	3.24

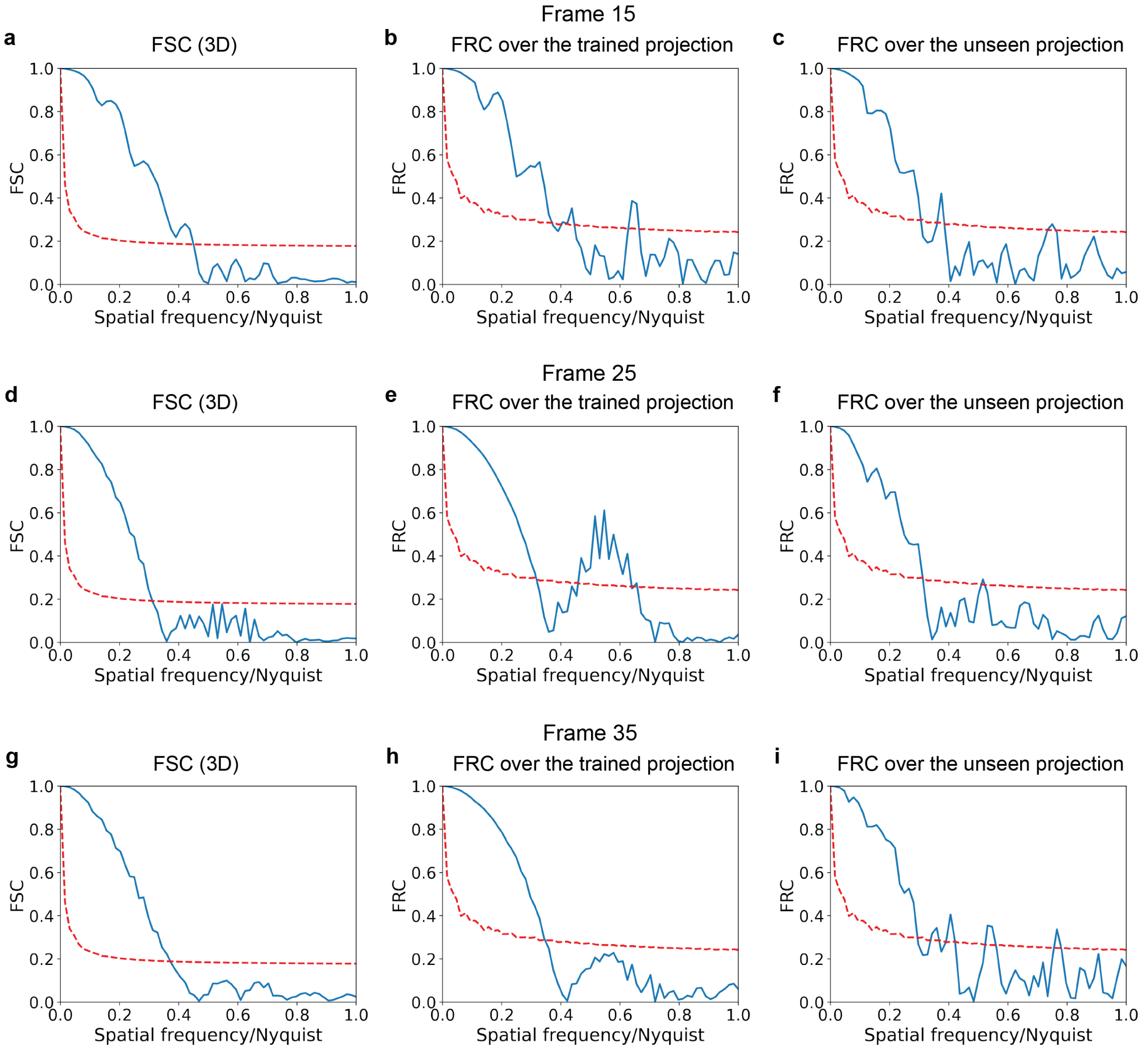

Supplementary Figure 4: Demonstration of the retrieved resolution for the 4D-ONIX reconstructions trained with 16 experiments of quasi-reproducible processes. The blue curves represent the 3D Fourier Shell Correlation (FSC) and 2D Fourier Ring Correlation (FRC) across different spatial frequencies, while the red dashed curves indicate the half-bit criterion. Three example timestamps are shown. **a** shows the FSC between the 4D-ONIX reconstruction and the 3D ground truth for timestamp 15, which gives a resolution of around 4 voxels. **b** and **c** show for the same timestamp the 2D FRC for the seen projection and the unseen projection, respectively, which correspond to a resolution of 5 pixels and 6 pixels, respectively. **d** the FSC for timestamp 25, which gives a resolution of around 6 voxels. **e** and **e** the 2D FRC for the seen projection and the unseen projection for timestamp 25, which corresponds to a resolution of 6 pixels for both. **g** the FSC for timestamp 35, which gives a resolution of 5 voxels. **h** and **i** the 2D FRC for the seen projection and the unseen projection for timestamp 35, which corresponds to a resolution of 6 pixels and 7 pixels, respectively.

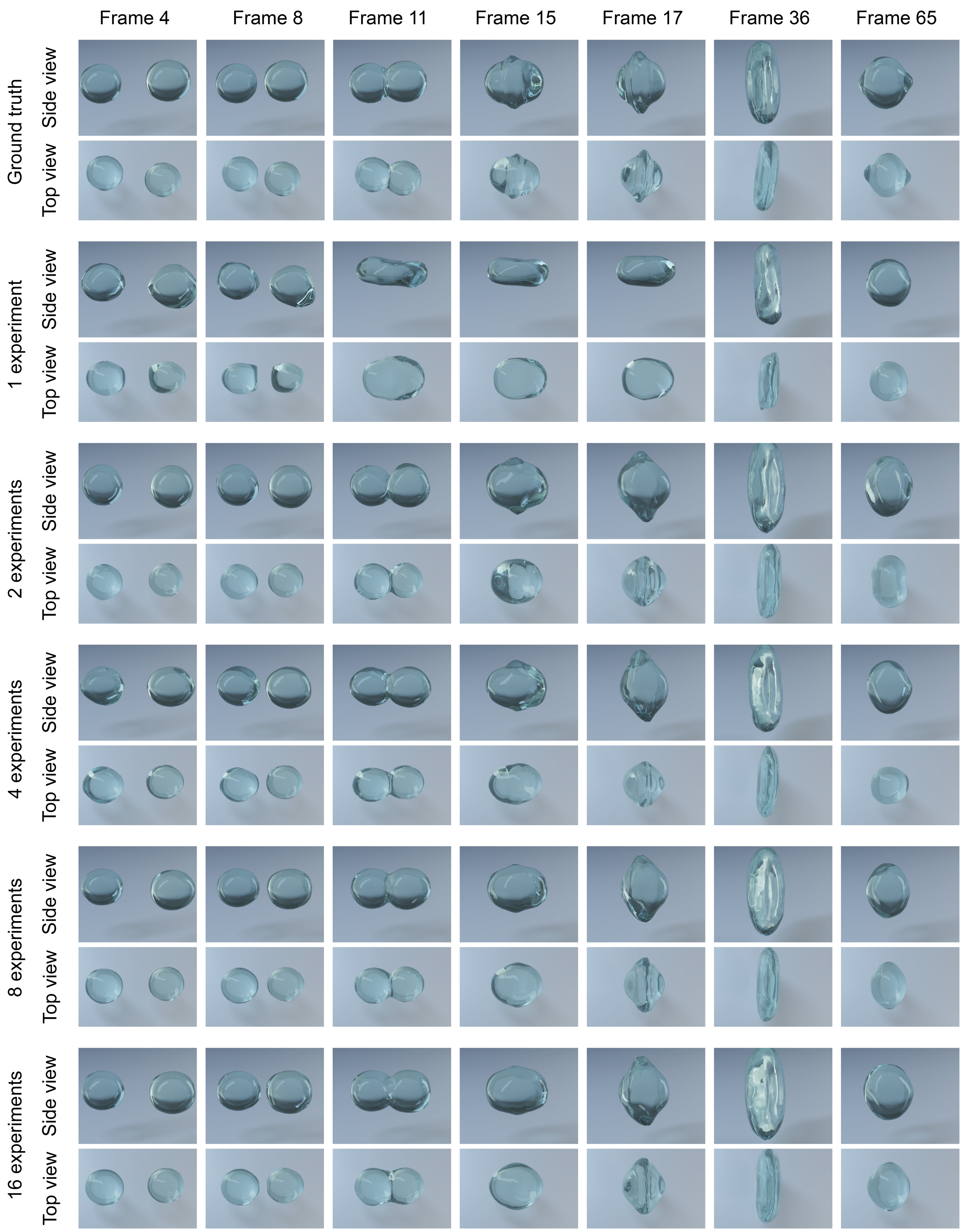

Supplementary Figure 5: Demonstration of 4D-ONIX reconstructions trained with different numbers of experiments for quasi-reproducible processes. For the selected timestamps, the 3D rendering from the side and top views of the ground truth and the 4D-ONIX reconstructions using different numbers of experiments are presented.

Supplementary Note 3: Data preparation and results of 4D-ONIX on experimental additive manufacturing data

As reported in the main article, we mimic multiple XMPI experiments using a time-resolved tomographic dataset of the remelting process of the magnetite-modified alumina. The original time-resolved tomographic dataset has the shape of $60 \times 200 \times 960 \times 180$, where 60 is the number of time stamps, 200 is the number of projections, and 960×180 is the dimension of the measured projection. We first applied flat-field correction to remove the flat-field noise of the projections, and next applied a Paganin filter to enhance the contrast and mitigate the noise on the measured intensity images [5]. Then, we selected a 960×64 region where the remelting dynamics happen and resized the selected area to 128×64 for faster computation.

For each tomogram, a 3D "ground truth" was prepared using the gridrec tomographic reconstruction algorithm [6] on the full set of 200 projections. This ground truth serves as a reference to validate 4D-ONIX's performance.

To train 4D-ONIX, we simulated multiple XMPI experiments by extracting ultra-sparse projections from the tomographic dataset. As shown in Figure 6, we selected three projections per experiment, where the geometry is similar to the XMPI setup reported in [7]. The angle of the first projection is denoted as φ_1, consistent with the notation used in Figure 2**a** of the main article. The relative angles between the first and second projections and between the second and third projections are represented by $\Delta\varphi_1$ and $\Delta\varphi_2$, respectively. In this case, we use $\Delta\varphi_1 = \Delta\varphi_2 = 27°$. For instance, if $\varphi_1 = 0$, the three projection angles are 0°, 27°, and 54°. We evaluated the performance of 4D-ONIX trained with different numbers of experiments, ranging from 1, 2, 4, 8, 16, to 32. The φ_1 angles of the selected experiments are reported in Table 3, along with the 4D MSE and DSSIM values comparing the ground truth and the 4D-ONIX reconstructions. The evolution of 3D MSE and DSSIM values over time is presented in Figure 7.

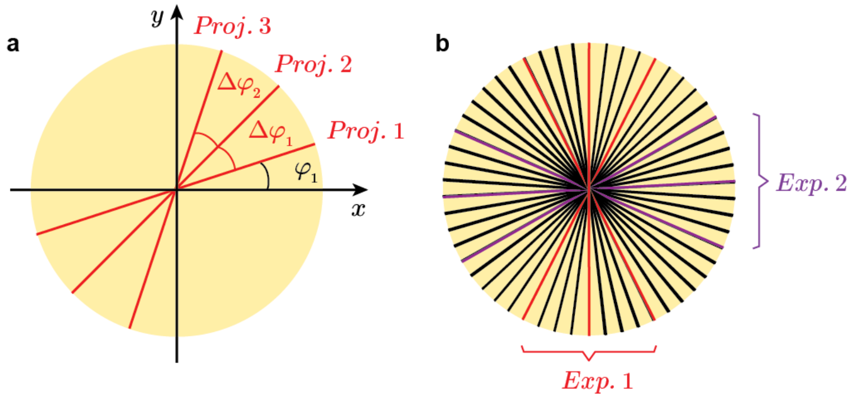

Supplementary Figure 6: **a** Geometry of the simulated X-ray multi-projection imaging (XMPI) experiment. Three projections (red lines) were selected from the tomographic dataset to replicate an XMPI measurement, with equal angular intervals of $\Delta\varphi_1 = \Delta\varphi_2 = 27°$. **b** Multiple XMPI experiments can be simulated using the 200 projections from the tomographic dataset. In this example, we demonstrate two experiments (colored red and purple), each consisting of three projections, separated by 27°. These experiments are treated as independent, and thus, the relative angles between them are not used as prior information.

Supplementary Note 4: Comparison with state-of-the-art methods

In this section, we evaluate the performance of 4D-ONIX by comparing it with two other reconstruction approaches: (i) simultaneous algebraic reconstruction technique (SART) [8], a classic iterative approach for sparse-view reconstructions, and (ii) Noise2inverse [9], a state-of-the-art deep-learning approach for sparse-view tomographic reconstruction.

We use the experimental additive manufacturing data for the evaluation. Since these methods lack

Supplementary Table 3: Comparison of 4D-ONIX reconstructions trained with different numbers of experiments for experimental additive manufacturing data.

# Experiments	φ_1 angles	MSE $\times 10^{-3}$	DSSIM $\times 10^{-2}$
1	0°	10.6	14.2
2	0°, 64.8°	10.2	9.7
4	0°, 32.4°,64.8°,97.2°	8.8	8.4
8	0°, 16.2°,32.4°, 48.6°, 64.8°, 81°,97.2°, 113.4°	7.4	9.5
16	0°, 8.1°, 16.2°, 24.3°, 32.4°, 40.5°, 48.6°, 56.7°, 64.8°, 72.9°, 81°, 89.1°, 97.2°, 105.3°, 113.4°, 121.5°	5.1	6.9
32	0°, 3.6°, 7.2°, 10.8°, 14.4°, 18°, 21.6°, 25.2°, 28.8°, 32.4°, 36°, 39.6°, 43.2°, 46.8°, 50.4°, 54° 71.1°, 74.7°, 78.3°, 81.9°, 85.5°, 89.1°, 92.7°, 96.3°, 99.9°, 103.5°, 107.1°, 110.7°, 114.3°, 117.9°, 121.5°, 125.1°	5.2	6.9

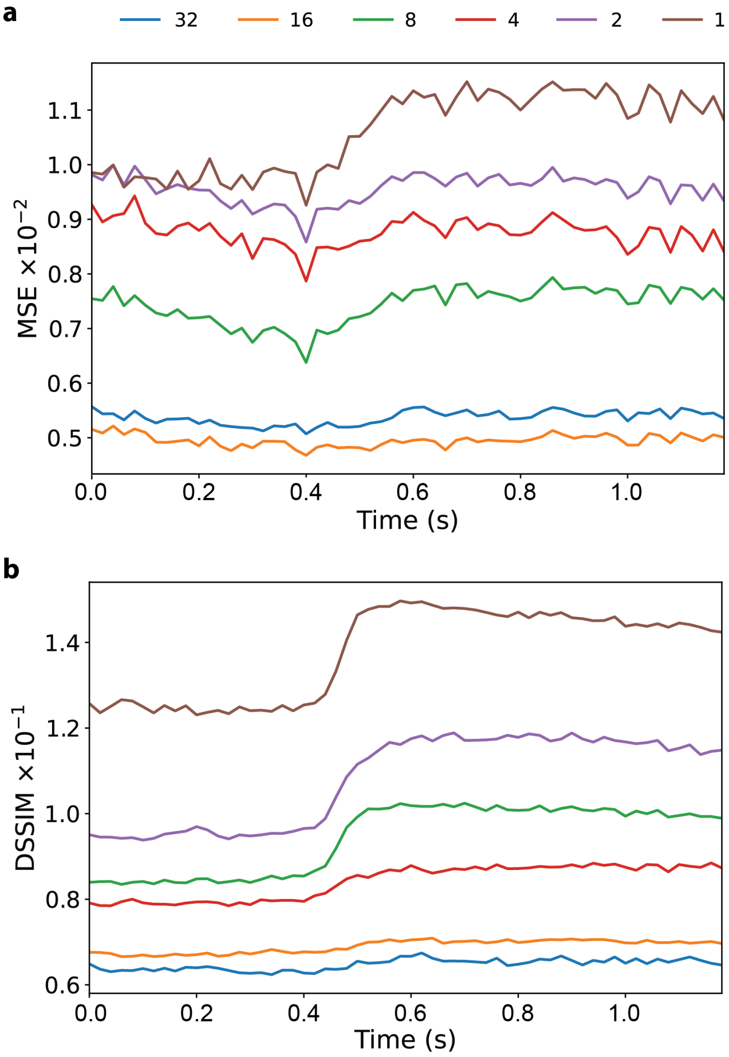

Supplementary Figure 7: Comparison of **a** Mean Squared Error (MSE) and **b** Dissimilarity Structure Similarity Index Metric (DSSIM) distribution with time for 4D-ONIX reconstructions trained with different numbers of experiments for the experimental additive manufacturing data. At each time point, the MSE and DSSIM were calculated by measuring the differences between the 4D-ONIX reconstructions and the 3D ground truth generated using the gridrec tomographic reconstruction algorithm with the full set of 200 projections.

the capacity to generalize across different experiments, we evaluate using a hypothetical scenario where more projections were used to reconstruct 4D from a single experiment. To have a fair comparison, we also show the results of 4D-ONIX trained by using more projections of a single experiment, as denoted by 4D-ONIX*. The results are shown in Figure 8 and Figure 9.

In Figure 8, the orange lines and the bottom x-axis show the results of SART, Noise2inverse, and 4D-ONIX*, reconstructed using 3, 6, 12, and 24 projections for a single 4D experiment. The blue lines and the upper x-axis represent the results of 4D-ONIX, reconstructed by combining different numbers of experiments, each with three projections, as summarized in Table 3. Figure 9 shows 3D renderings of the reconstructions from different methods alongside the ground truth. To achieve optimal visualization, a specific threshold was applied to the rendering of each method. As illustrated in Figures 8 and 9, 4D-ONIX trained with 32 experiments achieves comparable performance to SART, Noise2inverse, and 4D-ONIX* trained on single experiments with 24 projections. However, all reconstructions exhibit artifacts due to the noisy nature of the experimental projections.

References

[1] Wang, Z., Bovik, A. C., Sheikh, H. R. & Simoncelli, E. P. Image quality assessment: From error visibility to structural similarity. *IEEE Transactions on Image Processing* **13**, 600–612 (2004).

[2] Van der Walt, S. *et al.* scikit-image: image processing in python. *PeerJ* **2**, e453 (2014).

[3] Saxton, W. O. & Baumeister, W. The correlation averaging of a regularly arranged bacterial cell envelope protein. *Journal of Microscopy* **127**, 127–138 (1982).

[4] Van Heel, M. & Schatz, M. Fourier shell correlation threshold criteria. *Journal of Structural Biology* **151**, 250 – 262 (2005).

[5] Paganin, D., Mayo, S., Gureyev, T., Miller, P. & Wilkins, S. Simultaneous phase and amplitude extraction from a single defocused image of a homogeneous object. *Journal of microscopy* **206**, 33–40 (2002).

[6] Dowd, B. A. *et al.* Developments in synchrotron x-ray computed microtomography at the national synchrotron light source. In *Developments in X-ray Tomography II*, vol. 3772, 224–236 (SPIE, 1999).

[7] Asimakopoulou, E. M. *et al.* Development towards high-resolution khz-speed rotation-free volumetric imaging. *Optics Express* **32**, 4413–4426 (2024).

[8] Andersen, A. H. & Kak, A. C. Simultaneous algebraic reconstruction technique (sart): a superior implementation of the art algorithm. *Ultrasonic imaging* **6**, 81–94 (1984).

[9] Hendriksen, A. A., Pelt, D. M. & Batenburg, K. J. Noise2inverse: Self-supervised deep convolutional denoising for tomography. *IEEE Transactions on Computational Imaging* **6**, 1320–1335 (2020).

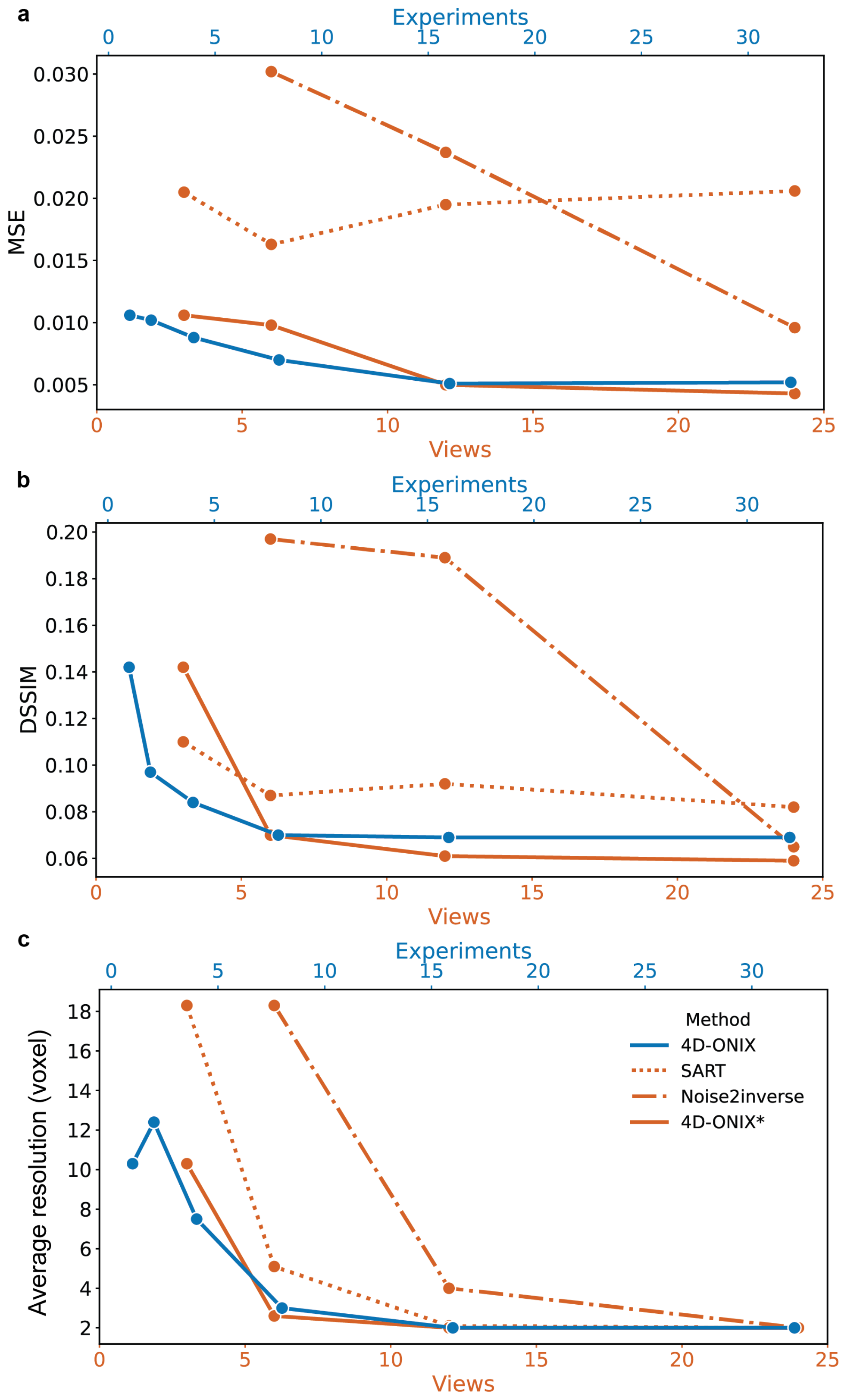

Supplementary Figure 8: Comparison of **a** Mean Squared Error (MSE), **b** Dissimilarity Structure Similarity Index Metric (DSSIM), and **c** average resolution determined by Fourier Shell Correlation (FSC) for the reconstructions of experimental additive manufacturing data using different methods. The bottom x-axis (orange) and orange lines represent the results of Simultaneous Algebraic Reconstruction Technique (SART), Noise2inverse, and 4D-ONIX*, reconstructed with varying numbers of projections. The upper x-axis (blue) and blue lines represent the results of 4D-ONIX, reconstructed by combining different numbers of experiments, each using three projections. Note that the first data points of 4D-ONIX and 4D-ONIX* represent the same experiment, as 4D-ONIX* trained with three projections corresponds to 4D-ONIX trained with a single experiment. The MSE and DSSIM were calculated in 4D by comparing the difference between the 4D-ONIX reconstructions and the ground truth reconstructed using 200 projections.

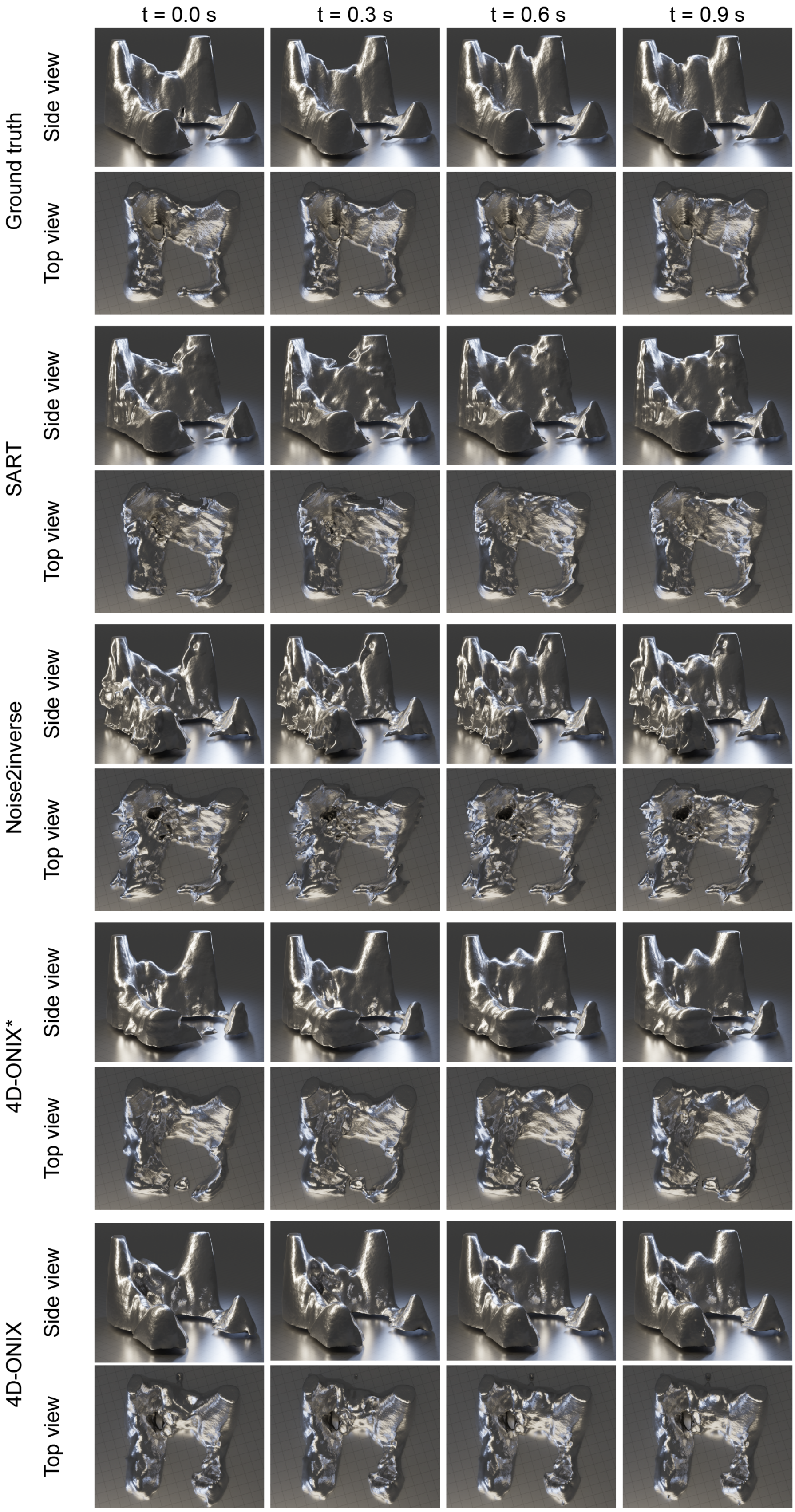

Supplementary Figure 9: Demonstration of 4D-ONIX reconstructions comparing with Simultaneous Algebraic Reconstruction Technique (SART), Noise2inverse, 4D-ONIX*, and ground truth. For the selected time points, the 3D rendering of the reconstructed results from the side and top views are presented. The results of SART, Noise2inverse, and 4D-ONIX* are reconstructed using 24 projections. The results of 4D-ONIX are reconstructed by combining 32 experiments of 3 projections.
