# Supplementary material for: 4D-ONIX for reconstructing 3D movies from sparse X-ray projections via deep learning
Source: Commun Eng. 2025 Mar 21;4:54. doi: 10.1038/s44172-025-00390-w (PMC11928503; doi:10.1038/s44172-025-00390-w)
Supplement: Supplementary file 2 — Description of Additional Supplementary Files [file 44172_2025_390_MOESM2_ESM.pdf]

Description of Additional Supplementary Files

File name: Supplementary Movie 1

Description: Reconstruction results of reproducible water droplet collisions (side view)

File name: Supplementary Movie 2

Description: Reconstruction results of reproducible water droplet collisions (top view)

File name: Supplementary Movie 3

Description: Reconstruction results of quasireproducible water droplet collisions (side view)

File name: Supplementary Movie 4

Description: Reconstruction results of quasireproducible water droplet collisions (top view)

File name: Supplementary Movie 5

Description: Reconstruction results from experimental additive manufacturing data
